# *Lactobacillus plantarum* and *Bifidobacterium longum* Alleviate Liver Injury and Fibrosis in Mice by Regulating NF-κB and AMPK Signaling

**DOI:** 10.4014/jmb.2310.10006

**Published:** 2023-12-26

**Authors:** Dong-Yun Lee, Jung-Woo Shin, Yoon-Jung Shin, Seung-Won Han, Dong-Hyun Kim

**Affiliations:** 1 Neurobiota Research Center, College of Pharmacy, Kyung Hee University, Seoul 02447, Republic of Korea; 2 PB Department, NVP Healthcare, Inc., Suwon 16209, Republic of Korea

**Keywords:** Live biotherapeutic product, liver injury, liver fibrosis, NF-κB activation, AMPK activation

## Abstract

In a preliminary study, live biotherapeutic products (LBPs) *Lactobacillus plantarum* LC27 and *Bifidobacterium longum* LC67 inhibited the secretion of alanine transaminase (ALT) and aspartate transaminase (AST) in LPS-stimulated HepG2 cells, while *Escherichia coli* K1 (Ec) increased ALT and ALT secretion. Therefore, we examined the effects of LC27 and LC67 on LPS-induced liver injury and fibrosis in mice and the correlation between their biomarkers in cell and animal experiments. Orally administered LC27 or LC67 significantly decreased blood ALT, AST, γ-glutamyl transferase (γGTP), TNF-α, triglyceride (TG), total cholesterol (TCh), total bile acid, and LPS levels, liver TNF-α, toll-like receptor-4 gene (*Tlr4*), α-smooth muscle actin (αSMA), and collagen-1 expression and αSMA^+^GFAP^+^ and NF-κB^+^F4/80^+^ cell populations, and colonic *Tlr4*, TNF-α, and IL-6 expression and NF-κB-positive cell population in LPS-treated mice. Furthermore, they increased AMPKa phosphorylation in the liver and colon. However, Ec increased the expression of TNF-α and IL-6 in blood, liver, and colon. The suppression of LPS-stimulated ALT and AST secretion in HepG2 cells by LBPs was positively correlated with their ameliorating effects on LPS-induced blood γGTP, ALT, and AST levels and liver αSMA and collagen-1 expression in mice. Based on these findings, LC27 and LC67 may improve liver injury and fibrosis by regulating NF-κB and AMPK signaling pathway and a protocol that can assay the inhibitory activity of LBPs on LPS-induced ALT and AST secretion in HepG2 may be useful for guessing their antihepatitic effects in the in vivo experiments.

## Introduction

The gut is anatomically and physiologically connected with the liver [1]. Therefore, gut bacteria and/or their byproducts can be ceaselessly exposed to the liver through the gut [1-4]. Stressor-induced gut dysbiosis increases the production of gut bacterial endotoxins including lipopolysaccharide (LPS) [4-6]. Excessive exposure to LPS induces liver damage with gut inflammation by activating NF-κB signaling, which proceeds to liver inflammation and fibrosis [7, 8].

Live biotherapeutic products (LBPs), including lactobacilli and bifidobacteria, inhibit the infection to pathogens [9-11], modulate host immune function [12, 13], and alleviate alcohol-, high-fat diet-, or acetaminophen-induced liver injury [14-17]. *Lactobacillus rhamnosus* CCFM1107 alleviates ethanol-induced liver damage in mice [18]. *Lactobacillus* (*Lactiplantibacillus*) *plantarum* LC27 and *Bifidobacterium longum* LC67 alleviate ethanol-, high-fat diet-, or 2,4,6-trinitrobenzenesulfonic acid (TNBS)-induced liver injury in mice [19-21]. *Akkermansia muciniphila* attenuates acetaminophen-induced liver injury in mice by modulating gut microbiota [14]. *Lactobacillus helveticus* R0052 mitigates D-galactosamine-induced liver damage in rodents via the modulation of gut microbiota [22]. *Bifidobacterium lactis* TY-S01 ameliorates ethanol-induced liver injury in mice by modulating gut microbiota [23]. Probiotic VSL#3 also attenuates liver damage in rodents by suppressing intestinal permeability [24]. These findings suggest that LBPs may mitigate liver injury with increased gut permeability by modulating gut microbiota. Nevertheless, the action mechanism of probiotics against liver injury and fibrosis remains elusive.

In the preliminary study, LBPs *Lactobacillus plantarum* LC27 and *B. longum* LC67 inhibited LPS-stimulated alanine transaminase (ALT) and aspartate transaminase (AST) secretion in HepG2 cells. Therefore, to investigate whether these probiotics could alleviate LPS-induced liver injury in vivo, we examined the effects of LC27 and LC67 on LPS-induced liver injury and fibrosis in mice and the correlation between their biomarkers in cell and animal experiments.

## Materials and Methods

### Materials

Antibodies for α-smooth muscle actin (αSMA), F4/80, and CD11c were purchased from Invitrogen. Enzyme-linked immunosorbent assay (ELISA) kits for cytokines were bought in Ebioscience (USA). Fetal bovine serum (FBS) and 4',6-diamidino-2-phenylindole (DAPI) were bought in Sigma.

### Preparation of LC27 and LC67

LC27 (KCCM11801P) and LC67 (KCCM11802P) were isolated from kimchi and human bacteria collection, respectively. They were identified using gram staining, API kit, and 16S rRNA gene and whole genome sequencing, which were performed, as previously reported [25]. The genomes of LC27 (4 contigs) and LC67 (3 contigs) were 3,319,056 bp with a GC content of 44.5% and 2,369,489 bp with a GC content of 60.1%, respectively. Their whole genome sequences exhibited the highest phylogenetic similarity to *Lactiplantibacillus lactis* NCTC13644 (99.6%) and *B. longum* JCM1217 (99.2%), respectively, using OrthoANI (Fig. S1).

LC27 and LC67 were cultured in MRS broth or general media for probiotics at 37°C for 18 h and 24 h, respectively, centrifuged, freeze-dried, and suspended in phosphate buffered saline (PBS) and 1% trehalose for cell and animal experiments, respectively, as previously reported [21]. The number of viable LC27 and LC67 strains were determined by plate counting on MRS (BD, USA) and TOS-MU agars (MBcell, Republic of Korea), respectively.

### Culture of HepG2 Cells

HepG2 cells (Korean Cell Line Bank) were cultured in DMEM (Sigma) containing 1% antibiotic-antimycotic and 10% FBS at 37°C. The cells (2 × 10^6^ cells/ml) were treated with LC27 or LC67 (1 × 10^3^ and 1 × 10^6^ CFU/ml) in the presence or absence LPS (100 ng/ml) for 20 h. ALT, AST, and lactic dehydrogenase (LDH) levels were assayed using their assay kits.

### Animals

C57BL/6 mice (male, 19-21 g, 8 weeks-old) were made a purchase from Koatech Inc. (Republic of Korea), maintained in the plastic cage under controlled condition (temperature, 20-22°C; humidity, 50 ± 10%; light/dark cycle, 12 h). Mice were allowed free access to standard chow and water. Animal experiments were ethically performed according to the University Guideline for Laboratory Animals Care and Usage (Institutional Animal Care and Used Committee Approval No, KHUASP(SE)-23087).

The hepatoprotective activity of probiotics was investigated in mice with acute LPS-prompted liver injury. Briefly, LPS (250 mg/kg/day, purified from *Escherichia coli*, Sigma) were intraperitoneally injected daily for 5 days. LC27 (1×10^8^ and 1×10^9^ CFU/mouse/day), LC67 (1 × 10^8^ and 1 × 10^9^ CFU/mouse/day), *E. coli* (Ec, 1 × 10^9^ CFU/mouse/day), or obeticholic acid (Oca, 5 mg/kg/day) was orally administered daily for 5 days from 24 h after the final LPS treatment. Tested LBPs were suspended in vehicle (1% trehalose). Mice were sacrificed 20 h after the final treatment with test agents.

Bloods were immediately collected from carotid artery and their sera were prepared, as previously reported [19]. Livers and colons were collected for ELISA, immunoblotting, and histological examination.

### Determination of ALT, AST, γ-Glutamyl Transpeptidase (γGTP), and Total Bile Acid (TBA)

ALT, AST, and γGTP levels in sera and cell culture supernatants were measured using their assay kits (Asan Pharmaceutical, Republic of Korea). TBA level was assayed using its assay kit (Elabscience, USA). Cytotoxicity was assayed using LDH assay kit (Biomax, Republic of Korea). (USA).

### Determination of Triglyceride (TG), Total Cholesterol (TCh), and LPS Levels

TG and TCh levels in the blood and liver were determined using their assay kits (Asan Pharmaceutical). LPS (endotoxin) level was assayed in sera using a LAL assay kit (Cape Cod Inc., USA).

### ELISA and Immunoblotting

Liver and colon tissues were lysed in the RIPA lysis buffer containing 1% phosphatase inhibitor cocktail and 1 %protease inhibitor cocktail (RPP) on ice and centrifuged (12,000 g, 4°C, and 15 min).

For the immunoblotting, the supernatants of liver and colon lysates were applied to SDS-PAGE, transferred to nitrocellulose membrane, and incubated with antibodies. Antibody-bound proteins were incubated with horseradish peroxidase-conjugated antibodies (Santa Cruz Biotechnology, USA), and visualized with excellent chemiluminescent substrate detection kit (Elabscience) [26].

For the cytokine assay, the supernatants were transferred in 96-well plate and assayed using ELISA kits (Ebioscience) [25].

### Quantitative Real-Time Polymerase Chain Reaction (qPCR) analysis

Total RNA was purified from liver and colon tissues using Qiagen RNeasy mini kit (Qiagen, Germany) [25]. Isolated mRNA (2 μg) was reversely transcribed using a PrimeScript cDNA synthesis kit (Takara, Japan). PCR was performed using the Rotor-Gene Q 5plex Platform (Qiagen) with TB Green Premix Ex Taq II (Takara). Primer sequences were as follows: toll-like receptor-4 gene (*Tlr4*) forward 5’-AGTGGGTCA AGGAACAGAAGCA-3’, reverse 5’-CTTTACCAGCTCATTTCTCACC-3’; αSMA gene (*Acta2*) forward 5’-CTGACAGAGGCACCACTGAA-3’, reverse 5’-GAAGGAATAGCCACGCTCAG-3’; collagen-1 gene (*Col1a1*) forward 5’-TCCTCCAGGGATCCAACGA-3’, reverse 5’-GGCAGGCGGGAGGTCTT-3’; 18S rRNA gene (*Rna18s*) forward 5’-CTAACCCGTTGAACCCCATT-3’, reverse 5’-CCATCCAATCGGTAGTAGCG-3’.

### Immunofluorescence Staining

Liver and colon tissues were collected from mice perfused and post-fixed with paraformaldehyde and cryosectioned, as previously reported [27]. Sectioned tissues were washed with PBS, blocked with serum, incubated with antibodies (for NF-κB, F4/80, αSMA, GFAP, and/or CD11c, Cell signaling Technology) for 12 h, then incubated with Alexa Fluor 488- or Alexa Fluor 594-conjuaged antibody (Invitrogen, USA) for 2 h. Nuclei were stained with DAPI. The sections were observed using a confocal microscope.

### Statistics

Data are expressed as mean ± SD using GraphPad Prism 9. Significance was analyzed using one-way ANOVA followed by Dunnett’s multiple range test (*p* < 0.05).

## Results

### LC27 and LC67 Decreased LPS-Induced AST and ALT Levels in HepG2 Cells

**Fig. 1:**
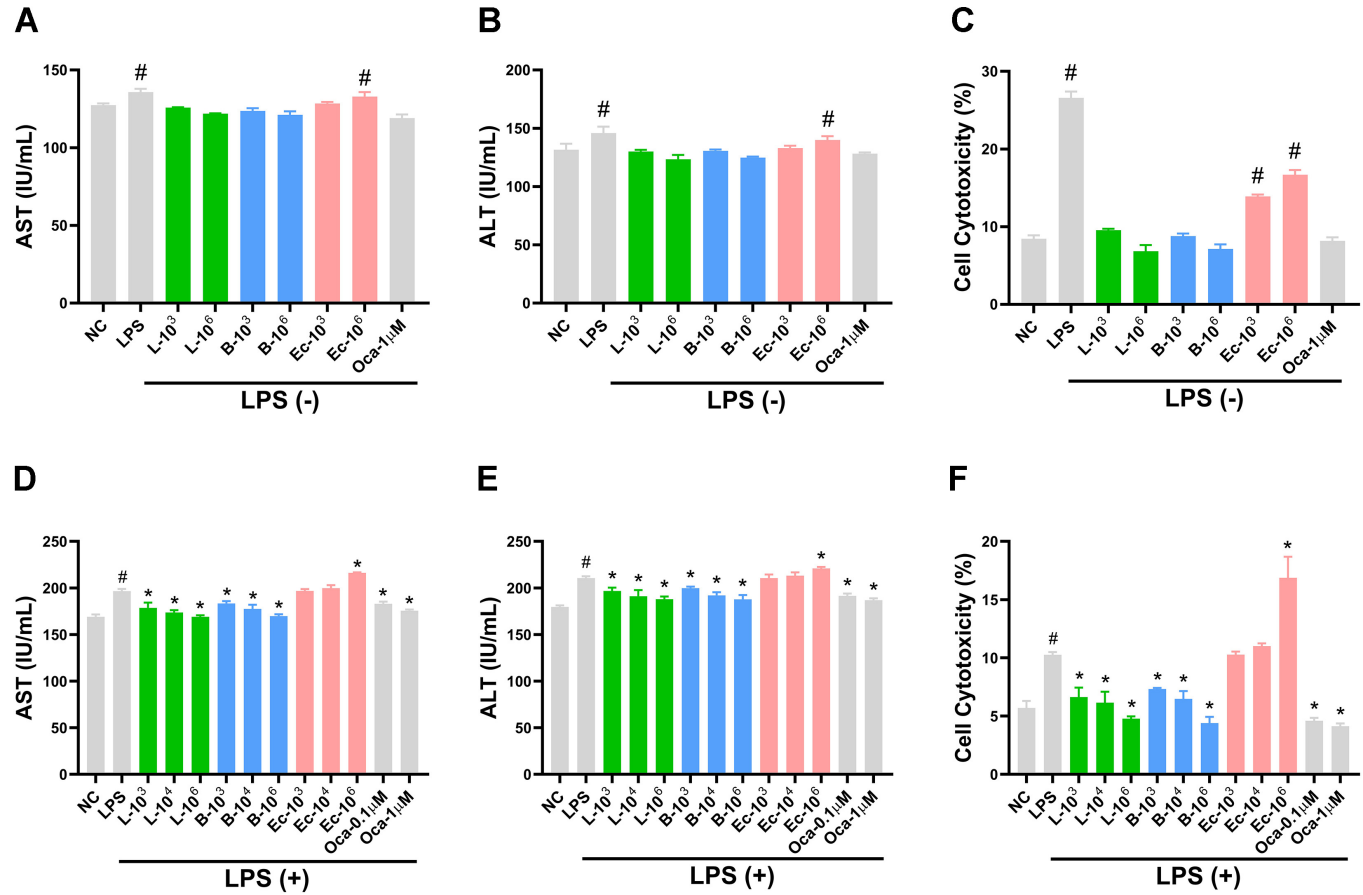
Effects of LC27, LC67, Ec, and Oca on AST and ALT and cytotoxicity in HepG2 cells stimulated with or without LPS. Effects on AST (**A**) and ALT (**B**) levels and cytotoxicity (**C**) in HepG2 cells. Effects on AST (**D**) and ALT (**E**) levels and cytotoxicity (**F**) in LPS-stimulated HepG2 cells. LBPs (L-10^3^, 1 × 10^3^ CFU/ml of LC27; L-10^6^, 1 × 10^6^ CFU/ml of LC27; B-10^3^, 1 × 10^3^ CFU/ml of LC67; B-10^6^, 1 × 10^6^ CFU/ml of LC67: suspended in PBS) were treated in HepG2 cells treated with or without LPS (100 ng/ml). NC and LPS were treated with vehicle (PBS) instead of LBPs. Biomarkers were assayed using their assay kits. ^#^*p* < 0.05 vs NC. *p < 0.05 vs LPS.

First, the effects of LC27, LC67, Ec, and Oca on AST and ALT levels were investigated in HepG2 cells treated with or without LPS. LC27, LC67, and Oca did not affect AST and ALT levels and cytotoxicity against HepG2 cells (Fig. 1A-1C). However, Ec increased AST and ALT secretion in HepG2 cells. Furthermore, Ec caused cytotoxicity against HepG2 cells.

Treatment with LPS significantly increased the cytotoxicity (Fig. 1D-1F). However, LC27, LC67 and Oca decreased dose-dependently LPS-induced AST and ALT secretion in HepG2 cells, while Ec increased it. LC27, LC67, and Oca protected against LPS-induced cytotoxicity of HepG2 cells, while Ec deteriorated it.

### LC27 and LC67 Alleviated Liver Damage and Colitis in Mice Treated with LPS

**Fig. 2:**
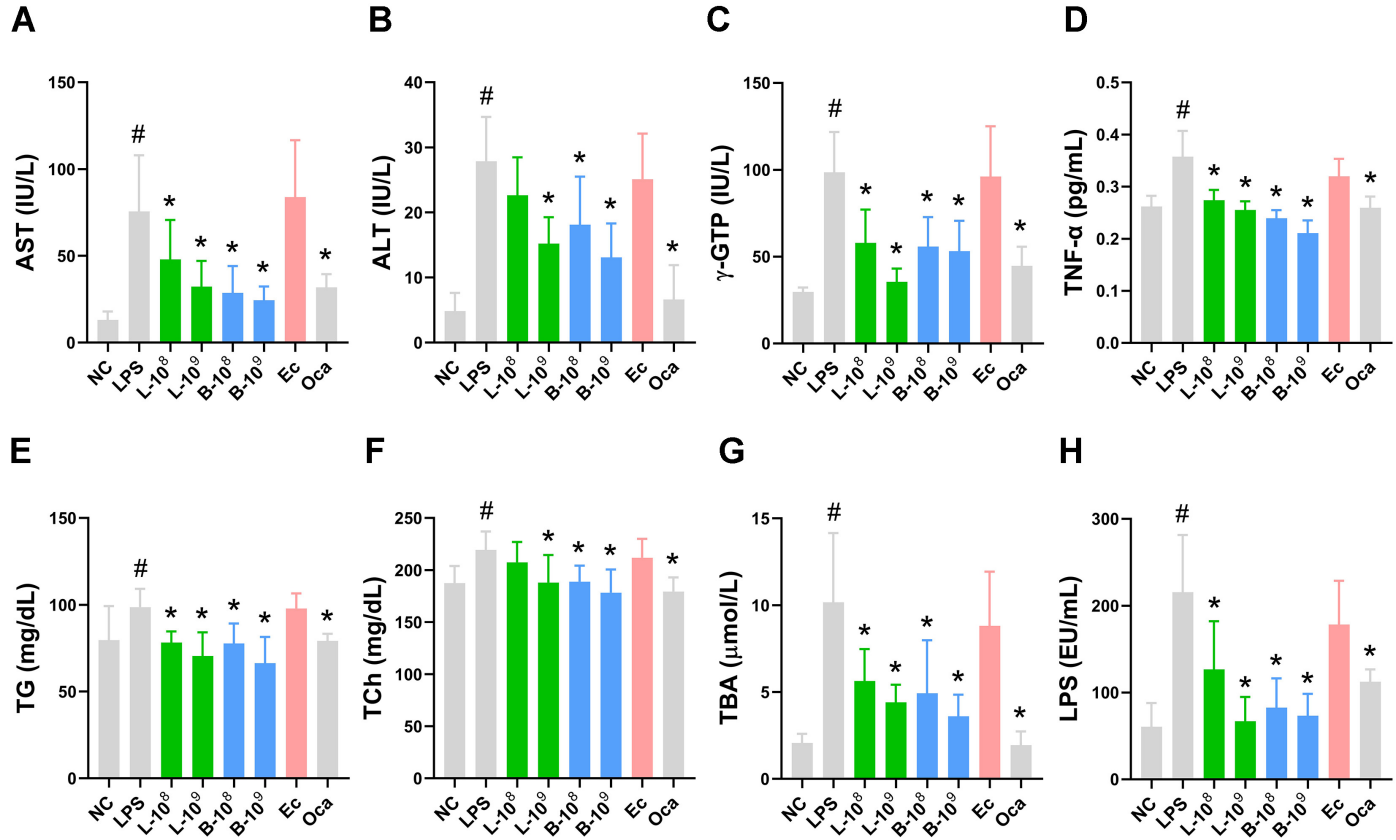
Effects of LC27 and LC67 on LPS-induced blood AST (A), ALT (B), γGTP (C), TNF-α (D), TG (E), TCh (F), TBA (G), and LPS levels (H) in mice treated with LPS. LBPs (L-8, 1×10^8^ CFU/mouse of LC27; L-9, 1 × 10^9^ CFU/mouse of LC27; B-8, 1 × 10^8^ CFU/mouse of NC67; B-9, 1 × 10^9^ CFU/mouse of LC67; Ec, 1 × 10^9^ CFU/mouse of *E. coli*; Oca, 5 mg/kg of Oca: suspended in 1% trehalose) were orally administered in mice once a day for 5 days. NC and LPS were treated with vehicle (1% trehalose) instead of LBPs. Biomarkers were determined in the blood using assay kits (**A-C, E**) and ELISA (**H**). ^#^*p* < 0.05 vs NC. *p < 0.05 vs LPS.

Next, the effects of LC27 and LC67 on LPS-induced liver damage and colitis were investigated in mice (Fig. 2). LPS treatment increased blood AST, ALT, γGTP, TNF-α, TBA, TG, and TCh levels. However, orally administered LC27 or LC67 significantly decreased LPS-induced blood AST, ALT, γGTP, TNF-α, TBA, TG, and TCh levels. Their effects are comparable to those of Oca. Interestingly, LC27 and LC67 decreased the LPS level in the blood, while Oca did not affect it. Ec treatment did not affect blood ALT, AST, and γGTP levels.

**Fig. 3:**
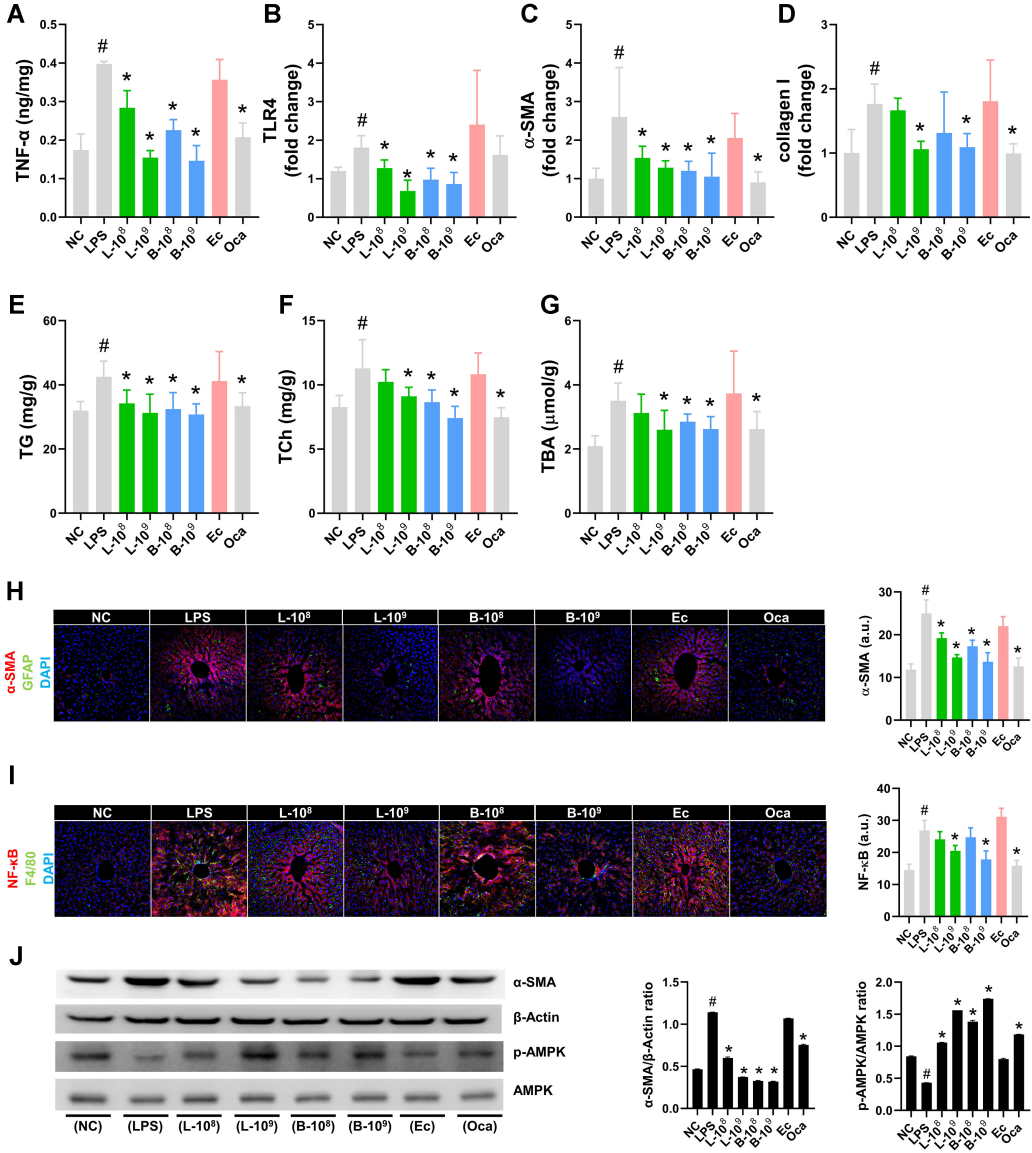
Effects of LC27 and LC67 on LPS-induced liver damage in mice. Effects on TNF-α (**A**), *Tlr4* (**B**), *Acta2* (**C**), and Col11a1 (**D**) expression, TG (**E**), TCh (**F**), and TBA (**G**) levels, αSMA^+^GFAP^+^ (**H**) and NF-κB^+^F8/40^+^ cell populations (**I**), and αSMA expression and AMPKa activation (**J**) in the liver of mice treated with LPS. LBPs (L-8, 1 × 10^8^ CFU/mouse of LC27; L-9, 1 × 10^9^ CFU/mouse of LC27; B-8, 1 × 10^8^ CFU/mouse of NC67; B-9, 1 × 10^9^ CFU/mouse of LC67; Ec, 1 × 10^9^ CFU/mouse of *E. coli*; Oca, 5 mg/kg of Oca: suspended in 1% trehalose) were orally administered in mice once a day for 5 days. NC and LPS were treated with vehicle [1% trehalose] instead of LBPs. Biomarkers were determined in the liver using ELISA (**A**), qPCR (**B-D**), assay kits (**E-G**), confocal microscope (**H, I**), and immunoblotting (**J**). ^#^*p* < 0.05 vs NC. *p < 0.05 vs LPS.

LPS treatment also increased TNF-α (assessed by ELISA), *Acta2* (αSMA gene), *Col1a1* (collagen-1 gene), and *Tlr4* expression (assessed by qPCR), αSMA^+^GFAP^+^ and NF-κB^+^F8/40^+^ cell populations, and TG, TCh, and TBA level in the liver (Fig. 3 and S2). However, orally administered LC27 or LC67 suppressed LPS-prompted TNF-α and TLR4 expression, αSMA^+^GFAP^+^ and NF-κB^+^F8/40^+^ cell numbers, and TG, TCh, and TBA levels in the liver. They also down-regulated LPS-prompted expression of αSMA, collagen-1, and their genes, assessed by immunoblotting and qPCR. Also, they increased LPS-suppressed AMPKa activation. Their effects (1 × 10^9^ CFU/mouse) were stronger compared to those of Oca (5 mg/kg). However, Ec deteriorated liver damage and fibrosis marker expression.

**Fig. 4:**
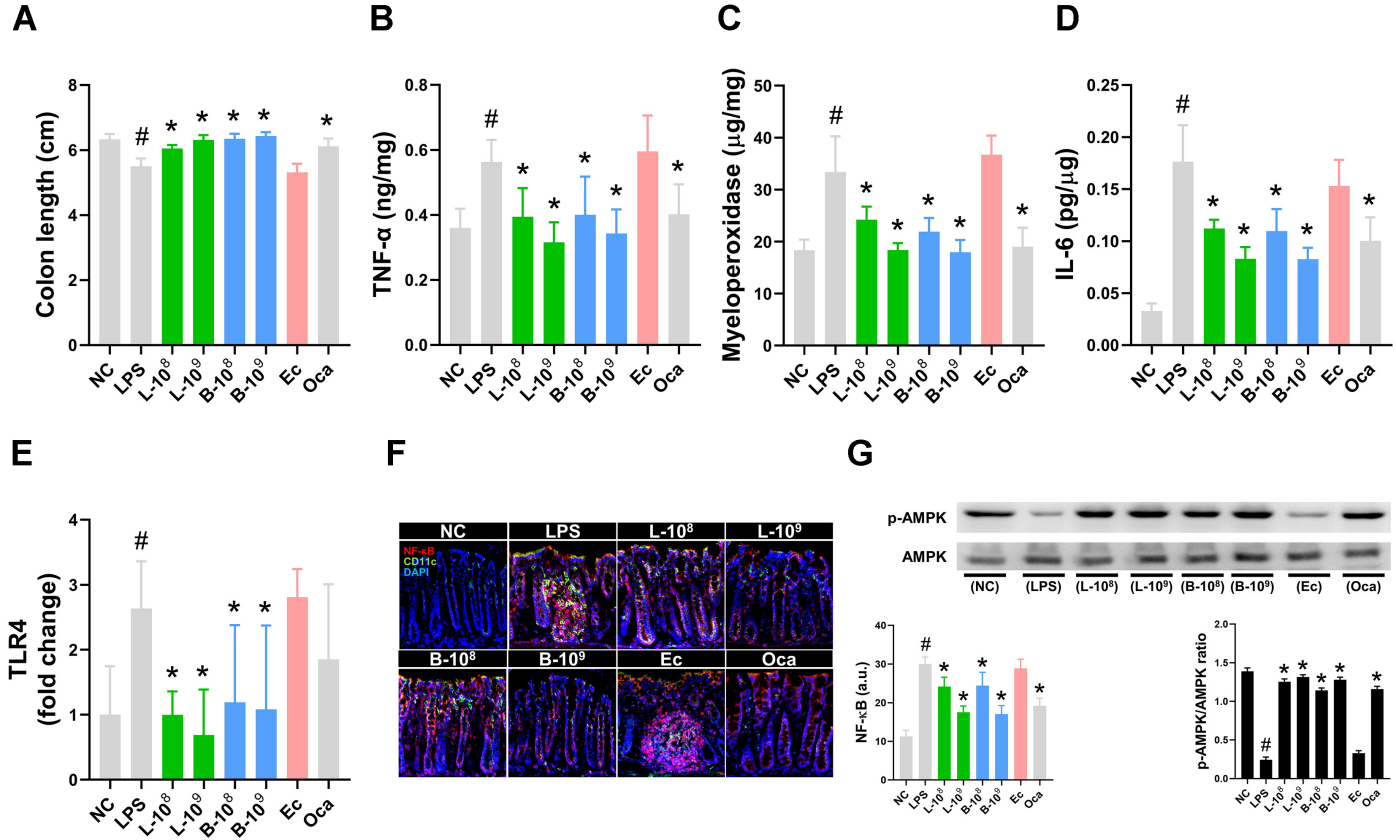
Effects of LC27 and LC67 on LPS-induced colitis in mice. Effects on colon length (**A**), TNF-α (**B**), myeloperoxidase (**C**), IL-6 (**D**), and *Tlr4* (**E**) expression (assessed by qPCR), NF-κB^+^CD11c^+^ cell population (**F**), and AMPKa activation (**G**) in the colon of mice treated with LPS. LBPs (L-8, 1 × 10^8^ CFU/mouse of LC27; L-9, 1 × 10^9^ CFU/mouse of LC27; B-8, 1 × 10^8^ CFU/mouse of NC67; B-9, 1 × 10^9^ CFU/mouse of LC67; Ec, 1 × 10^9^ CFU/mouse of *E. coli*; Oca, 5 mg/kg of Oca: suspended in 1% trehalose) were orally administered in mice once a day for 5 days. NC and LPS were treated with vehicle (1% trehalose) instead of LBPs. Biomarkers were determined in the colon using ELISA (**B-D**), qPCR (**E**), confocal microscope (**F**), and immunoblotting (**G**). ^#^*p* < 0.05 vs NC. *p < 0.05 vs LPS.

LPS treatment caused colon-shortening and up-regulated colonic myeloperoxidase, TNF-α, IL-6, and *Tlr4* expression and NF-κB^+^CD11c^+^ cell population (Fig. 4 and S3). Orally administered LC27 or LC67 attenuated colon shortening. They down-regulated LPS-induced colonic myeloperoxidase, TNF-α, IL-6, and *Tlr4* expression and NF-κB^+^CD11c^+^ cell number. They also induced AMPKa activation. Although Oca (5 mg/kg) suppressed myeloperoxidase, TNF-α, and IL-6 expression, its effect was weaker than those of LC27 and LC67 (1 × 10^9^ CFU/mouse). However, Ec deteriorated myeloperoxidase, TNF-α, IL-6, and *Tlr4* expression.

### Correlation between Liver Damage-Related Biomarkers in LPS-Treated HepG2 and Mice

**Fig. 5:**
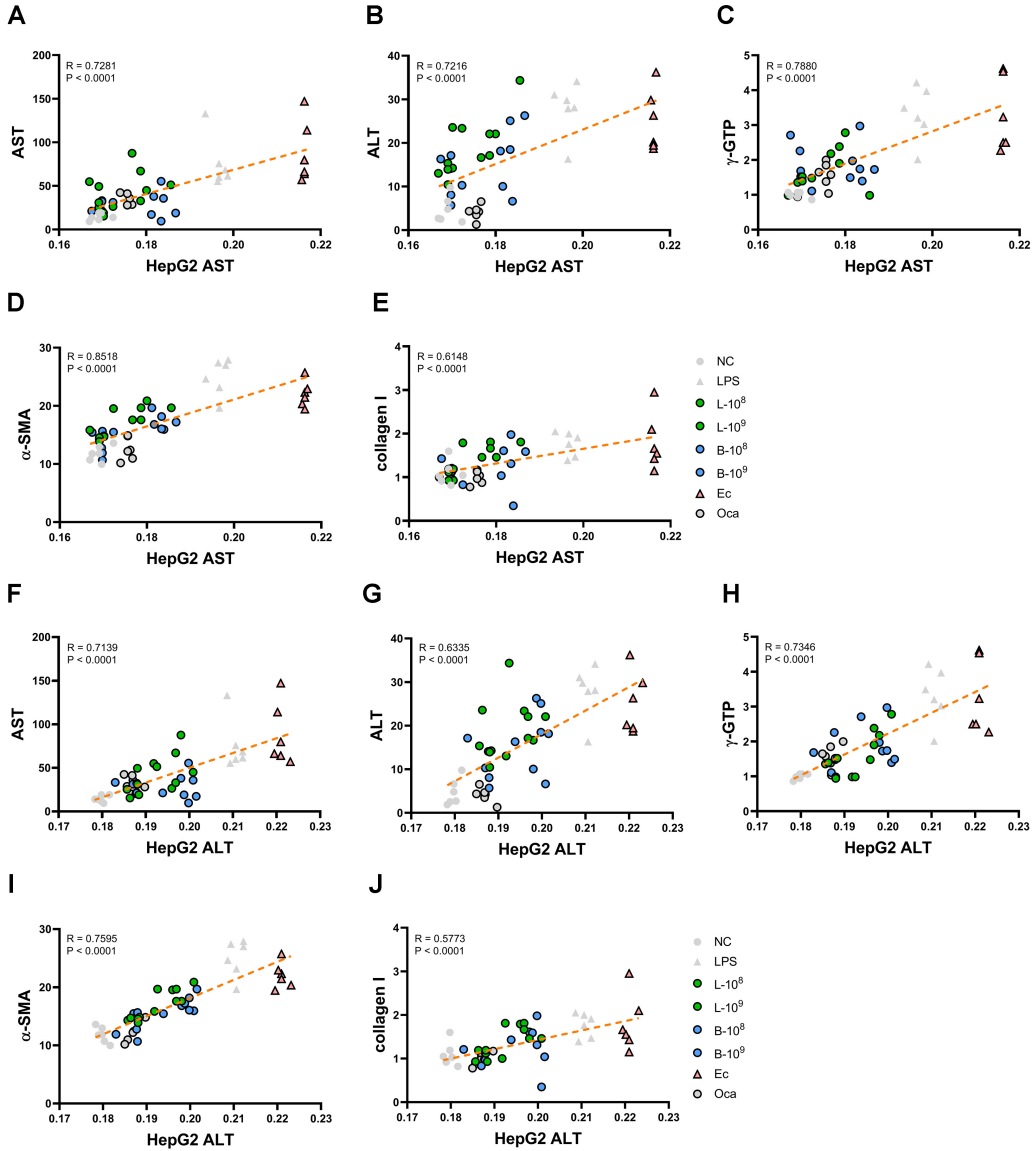
The correlation between liver damage-related biomarkers in LPS-stimulated HepG2 and those in mice with LPS-induced liver injury. The correlation between AST level in LPS-stimulated HepG2 cells and AST (**A**), ALT (**B**), γGTP (**C**), *Acta2* (**D**), or *Col1a1* (**E**) level in mice with LPS-induced liver injury. The correlation between ALT level in LPSstimulated HepG2 cells and AST (**F**), ALT (**G**), γGTP (**H**), *Acta2* (**I**), or *Col1a1* (**J**) level in mice with LPS-induced liver injury. The correlation was analyzed using the Pearson correlation coefficient.

To understand whether the biological activities of probiotics in the in vitro cell study could be proportional to their effects in the vivo animal study, we investigated the correlation between liver damage-related biomarkers in LPS-stimulated HepG2 and those in mice with LPS-prompted liver injury (Fig. 5). The suppression of LPS-stimulated ALT and AST levels in HepG2 cells by LC27 or LC67 was positively correlated with their inhibitory effects on blood ALT and AST levels (liver damage) and liver αSMA and collagen-1 levels (liver fibrosis) in mice with LPS-induced liver injury. In particular, the AST level in HepG2 cells showed the highest correlation with the liver αSMA level in mice, followed by blood γGTP levels. The ALT level in HepG2 cells showed the highest correlation with the liver αSMA level in mice, followed by blood γGTP levels.

## Discussion

Acute inflammation is the immediate response to detrimental stimuli, such as pathogens and their products, and may be a beneficial response [28-30]. However, excessive and/or prolonged exposure to pathogens and their byproducts such as endotoxins can cause severe inflammatory diseases such as inflammatory bowel disease and hepatitis [30-32]. Gut dysbiosis increases LPS level in the intestinal fluid, which induces gut inflammation with a leaky gut [33, 34]. The leaky gut accelerates the absorption of macromolecules such as LPS from the gastrointestinal tract into the blood and liver, resulting in systemic diseases including hepatitis and hepatic steatosis and fibrosis [6, 35].

We also found that excessive exposure of mice to LPS increased blood ALT, AST, γGTP, and TNF-α levels, liver NF-κB^+^F4/80^+^ cell number and TNF-α and *Tlr4* expression, and colonic TNF-α, myeloperoxidase, and *Tlr4* expression and NF-κB^+^CD11c^+^ cell number in mice. These findings explain that LPS may lead to hepatitis with gut inflammation by activating TLR4-mediated NF-κB signaling. The excessive exposure of mice to LPS increased liver αSMA and collagen-1 expression and TBA, TG, and TCh levels and blood TBA, TG, and TCh levels, while AMPKa activation in the colon and liver decreased. These findings explain suggest that excessive exposure to LPS may cause the initial stage of liver fibrosis with steatohepatitis by inducing αSMA and collagen-1 expression and by suppressing AMPKa signaling.

We also found that orally administered LC27 or LC67 (1 × 10^8^ and 1 × 10^9^ CFU/mouse) decreased LPS-prompted blood TNF-α and LPS levels, liver TNF-α and TLR4 expression and NF-κB-positive Kupffer’s cell number, and colonic TNF-α, myeloperoxidase, and *Tlr4* expression and NF-κB-positive cell number in mice. These findings explain that LC27 and LC67 may mitigate hepatitis with colitis by down-regulating TLR4-mediated NF-κB signaling. LC27 and LC67 suppressed LPS-prompted ALT, AST, and γGTP levels in the blood and αSMA and collagen-1 expression and αSMA-positive cell number in the liver. They decreased LPS-prompted TG, TCh, and TBA levels in the blood and liver. They increased AMPKa phosphorylation in the liver and colon. Furthermore, they suppressed ALT and AST levels in LPS-stimulated HepG2 cells and protected against LPS-induced cytotoxicity of HepG2 cells. The secretion of ALT and AST in HepG2 cells was significantly correlated with the cytotoxicity (Fig. S4). The suppression of LPS-stimulated ALT and AST secretion in HepG2 cells by LC27 or LC67 was positively correlated with their inhibitory effects on blood ALT and AST levels (liver damage), liver αSMA collagen levels (liver fibrosis), and liver TG and TC levels (steatohepatitis) in mice with LPS-prompted liver injury. These findings explain that LC27 and LC67 can alleviate liver damage and fibrosis and steatohepatitis through suppressing cytotoxicity and αSMA and collagen expression and inducing AMPK activation.

Many LBPs, including lactobacilli and bifidobacteria, alleviate alcohol-, high-fat diet-, or acetaminophen-induced liver injury in vivo [14-17]. LC27 and LC67 were previously reported to alleviate ethanol- or high-fat diet-prompted liver damage in mice by down-regulating bacterial LPS production and gut dysbiosis [19-21]. *Lactobacillus rhamnosus* CCFM1107 alleviates ethanol-prompted liver damages in mice with the regulation of gut microbiota [18]. *Akkermansia muciniphila* attenuates acetaminophen- or HFD/CCl4-induced liver damage by the modulation of gut microbiota [14, 36]. *L. helveticus* R0052 and *B. lactis* TY-S01 also mitigate D-galactosamine-prompted liver damage in mice via the modulation of gut microbiota [22, 23]. *LB. plantarum* Lp2 alleviates LPS-induced liver injury in mice by down-regulating NF-κB activation [37]. *L. casei* MYL01 alleviates ethanol-impaired hepatocytes in vitro by suppressing TLR4-mediated NF-κB activation [38]. We also found that in mice with LPS-induced liver injury, LC27 and LC67 suppressed the LPS level in the blood, and NF-κB-positive cell number and *Tlr4* and proinflammatory cytokine expression in the liver and colon. These findings explain that excessive exposure to LPS can cause hepatitis with colitis, which can be alleviated by LC27 and LC67 via the suppression of gut bacterial LPS production and NF-κB signaling.

## Conclusion

The suppression of LPS-stimulated ALT and AST secretion in HepG2 cells by LC27 or LC67 was positively correlated with their ameliorating effects on LPS-induced blood γGTP, ALT, and AST levels and liver αSMA, collagen-1, TNF-α, and *Tlr4* expression in mice. However, LC27 and LC67 up-regulated LPS-suppressed AMPK activation in the liver and colon. Conclusively, LC27 and LC67 may mitigate liver injury and fibrosis by suppressing NF-κB signaling and inducing AMPK signaling, and a protocol that can assay the inhibitory activities of LBPs on LPS-induced ALT and AST secretion in HepG2 may be useful for guessing their antihepatitic effects in the in vivo experiments.

## Supplemental Materials

Supplementary data for this paper are available on-line only at http://jmb.or.kr.
