# Supplementary material for: Lactobacillus plantarum and Bifidobacterium longum Alleviate Liver Injury and Fibrosis in Mice by Regulating NF-κB and AMPK Signaling
Source: J Microbiol Biotechnol. 2023 Dec 26;34(1):149–56. doi: 10.4014/jmb.2310.10006 (PMC10840473; doi:10.4014/jmb.2310.10006)
Supplement: Supplementary file 1 [file jmb-34-1-149-supple.pdf]

J. Microbiol. Biotechnol.
https://doi.org/10.4014/jmb.2310.10006

Lactobacillus plantarum and *Bifidobacterium longum* Alleviate Liver Injury and Fibrosis in Mice by Regulating NF-κB and AMPK Signaling

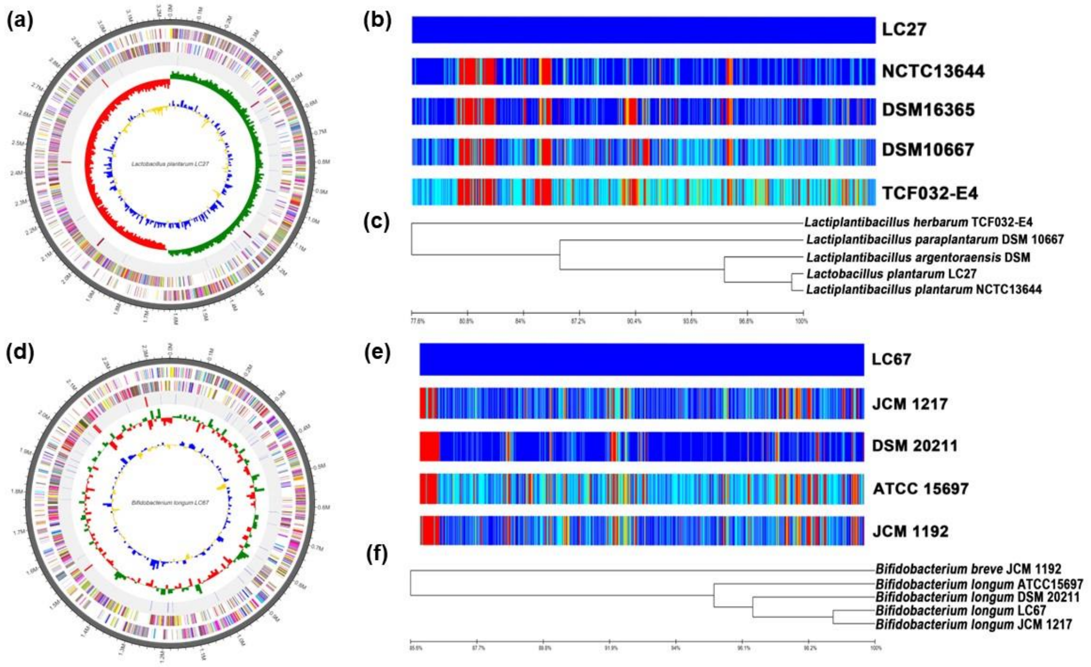

Figure S1. Taxonomic classification by genome-wide comparative analysis of LC27 and LC67. (a) The pseudochromosome drawn from 4 contigs for LC27. The outermost circle means contig. The inner circle is the color coded for the CDS information analyzed in the forward strand, and the inner circle is the CDS information analyzed in the reverse strand. The fourth circle from outside is tRNA (blue) and rRNA (red). The inner circle indicates GC skew metric information (green, higher than the average; red, lower than the average). The innermost circle is GC ratio metrics information (blue, higher values than average; yellow, lower values) GC skew and GC ratio metrics are displayed at 10kb intervals. (b) The pairwise ortholog matrix table (generated and colored according to the similarity between matching sequences) of LC27. (c) Neighbor-joining tree based on the OrthoANI distance matrix (analyzed by UPGMA dendrogram, Newick format) of LC27. (d) The pseudochromosome drawn from 3 contigs for LC67. (e) The pairwise ortholog matrix table (generated and colored according to the similarity between matching sequences). (f) Neighbor-joining tree based on the OrthoANI distance matrix of LC67.

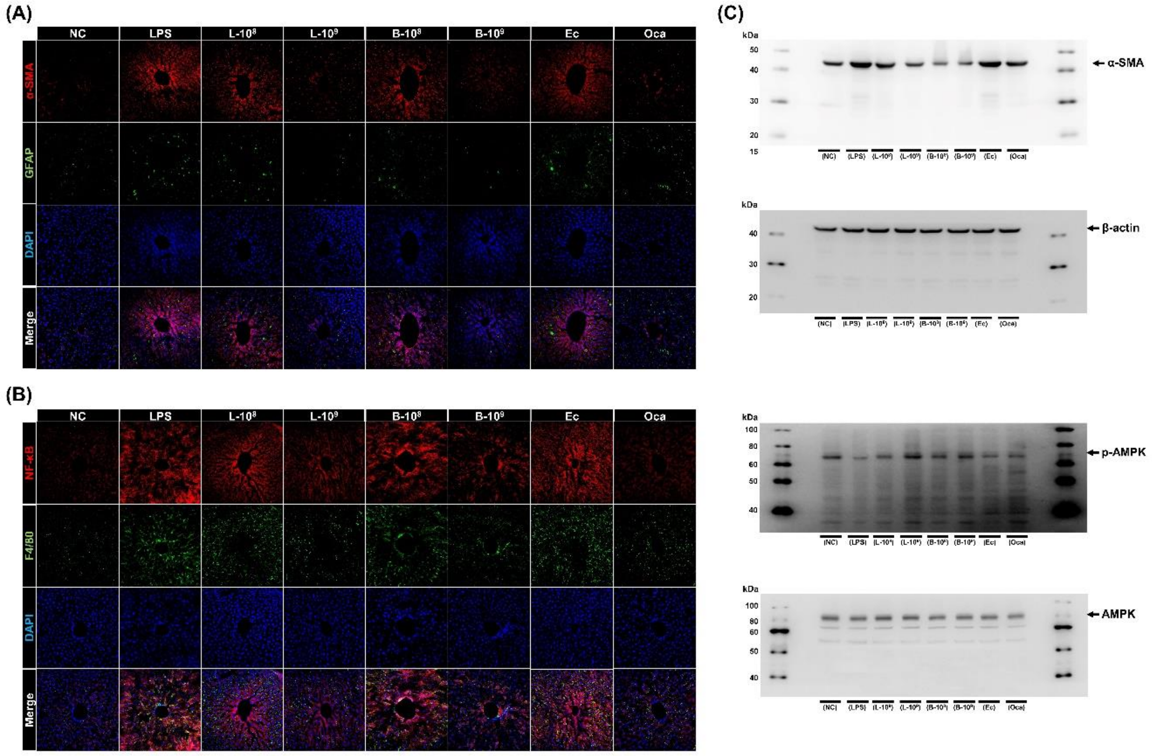

Figure S2. Effects of LC27 and LC67 on LPS-induced liver damage in mice. Effects on α-SMA$^+$GFAP$^+$ (A) and NF-κB$^+$F4/80$^+$ cell populations (B) and α-SMA expression and AMPKa activation (C) in the liver of mice treated with LPS.

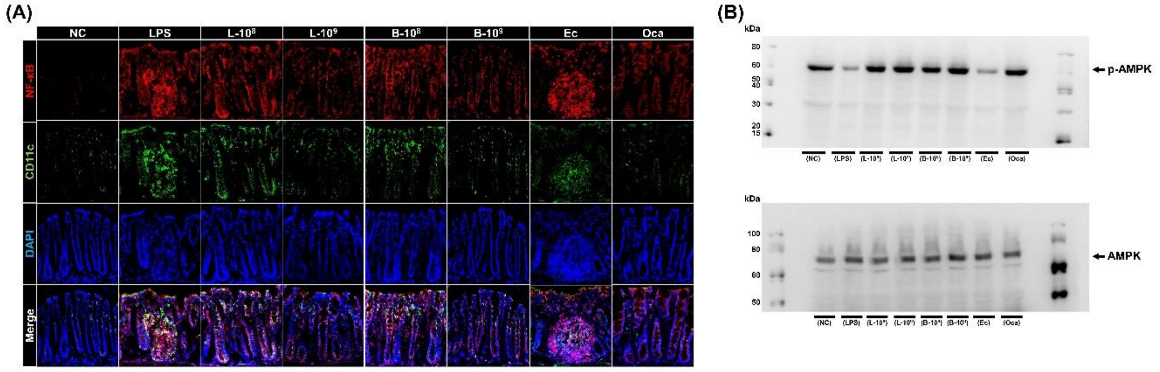

Figure S3. Effects of LC27 and LC67 on LPS-induced colitis in mice. Effects on NF-κB$^+$CD11c$^+$ cell population (A) and AMPKa activation (B) in the colon of mice treated with LPS.

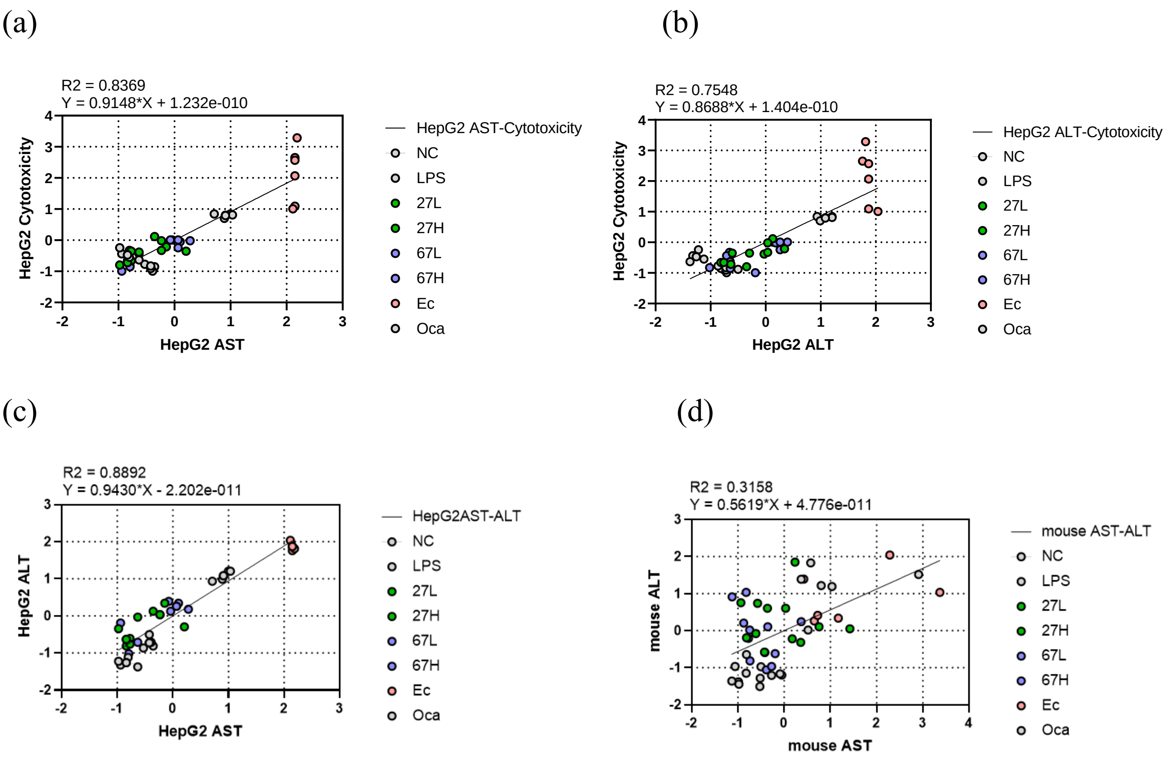

Figure S4. The correlation between liver damage-related biomarkers in LPS-stimulated HepG2 and those in mice with LPS-induced liver injury. (a) The correlation between AST and cytotoxicity in LPS-stimulated HepG2 cells. (b) The correlation between ALT and cytotoxicity in LPS-stimulated HepG2 cells. The correlation in (a) and (b) was analyzed using the Pearson correlation coefficient. (c) The correlation between AST and ALT levels in LPS-stimulated HepG2 cells. (d) The correlation between AST and ALT levels in mice with LPS-induced liver injury. The correlation in (c) and (d) was analyzed using analyzed by Q-Q plot.
